# 3D T1-weighted self-gated cardiac MRI for assessing myocardial infarction in mouse models

**DOI:** 10.1186/1532-429X-18-S1-Q49

**Published:** 2016-01-27

**Authors:** Xiaoyong Zhang, Hanwei Chen, Zijun Wei, Fei Yan, Shi Su, Yanchun Zhu, Bensheng Qiu, Xin Liu, Zhaoyang Fan, Guoxi Xie

**Affiliations:** 1 grid.59053.3a0000000121679639University of Science and Technology of China, Hefei, China; 2 grid.458489.c0000000104837922Shenzhen Institutes of Advanced Technology, Shenzhen, China; 3 Guangzhou Panyu Central Hospital, Guangzhou, China; 4 grid.50956.3f0000000121529905Cedars-Sinai Medical Center, Los Angeles, CA USA

## Background

Mouse models with mycardial infarction (MI) have been intensively used to investigate the cardiac remodeling and functional change [1,2]. MRI with ECG triggering and respiratory gating (or breath-holding) is commonly used to assess MI due to its noninvasive nature. However, the manipulations of external ECG triggering and respiratory gating are cumbersome due to the small size of mouse. To address this issue, a 3D self-gating (SG) MR technique with stack-of-stars sampling trajectories was proposed for retrospectively cardiac and respiratory-gated MI imaging in mouse models [3].

**Figure 1:**
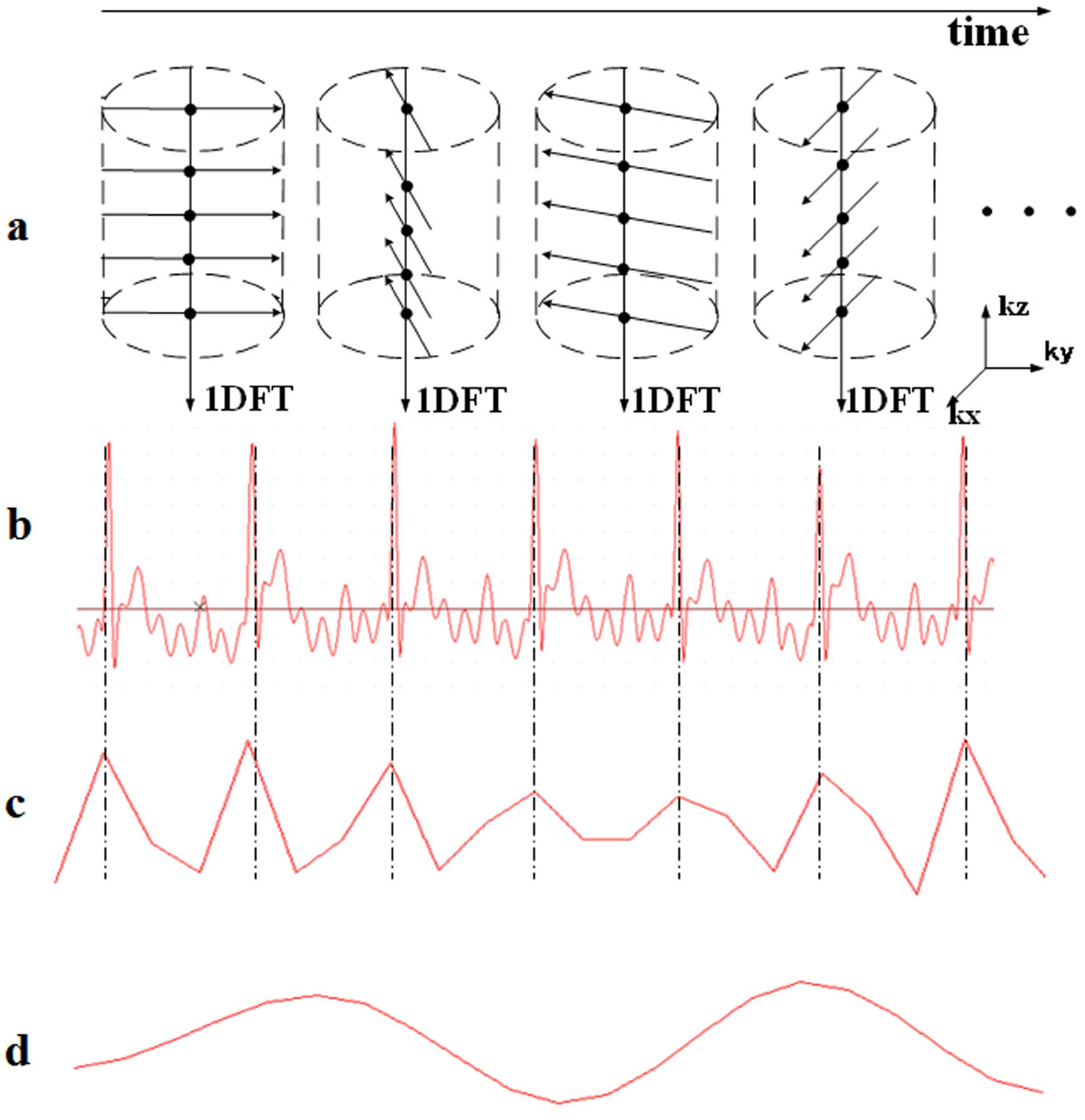
**Self-gating signals were compared with ECG signals**. (a) k-space sampling trajectories, the dot (•) denotes the SG data; (b) ECG signals recorded by the small animal ventilator; (c&d) cardiac and respiratory motions detected by the SG technique.

**Methods: Technical Design**: MR data was acquired by a T1-weighted GRE sequence with stack-of-stars sampling trajectories and a partition-first golden-angle reordering (Figure 1a). The centers of k-space lines acquired at the same angle from individual partitions were aggregated and used as an SG time point. The periodic respiratory and cardiac motions were then detected through an iterative filtering process on the SG time series according to the cardiac rate of 300~500 per minute and respiration rate of 70~110 per minute. After resorting the imaging data into appropriate cardiac and respiratory phases, motion-artifact-free were finally reconstructed.

**Experiments:** The SG technique was preliminarily validated on 5 mice with MI induction and all MR scans were performed on a 3T scanner (Siemens Tim Trio, Germany) with a customized 4-channal mouse coil. Typical imaging parameters for the ungated GRE sequence included: flip angle = 18°, TR = 4.2 ms, TE = 2.4 ms, spatial resolution = 0.6 × 0.6 × 1.5 mm^3^, bandwidth = 620 Hz/Pixel, partition number = 12, and a total number of 3200 projections were continuously collected, corresponding to a fixed scan time of 3 min. An amount of 0.5 ml gadolinium contrast agent with concentration of 0.5 mmol/ml (Consun Pharmaceutical Group Limited, GuangZhou, China) was injected to enhance the MI. The mice were sacrificed immediately after MRI for histological analysis and comparison to the MR results.

## Results

**Figure 2:**
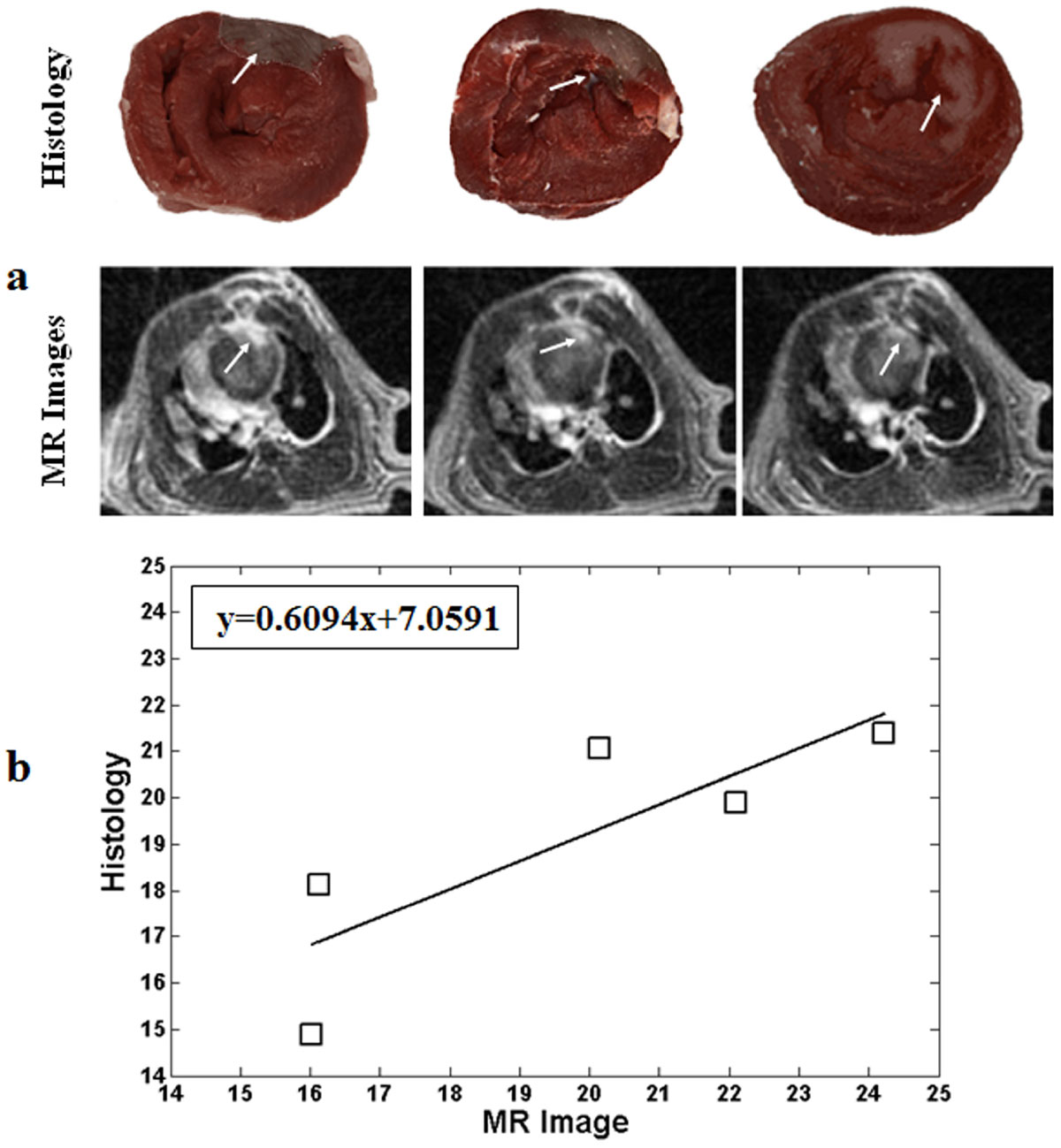
**(a) Representative histopathology pictures (top row) and reconstructed MR images (bottom row) from a mouse with MI induction**. The regions of MI (arrows) matched nicely between the histology and the proposed SG technique. (b) The correlation of the ratio of the infarction size and myocardial size between histology and the proposed method.

All MR scans were successfully conducted. The cardiac motion detected by the SG technique matched nicely to the ECG signals recorded by a small animal ventilator (Chengdu Taimeng Software Co.LTD, Chengdu, China) (Fig. 1b&1c). And the MI regions detected by the SG technique were also matched well to the histopathology analysis results (Fig. 2).

## Conclusions

A 3D SG technique was developed for assessing MI in mouse model. The preliminary in vivo study has demonstrated that the technique can correctly detect the MI, which may outperform the conventional MR techniques with ECG-triggering and respiratory gating.
